# Proton Pump Inhibitor Therapy before and after Endoscopic Submucosal Dissection: A Review

**DOI:** 10.1155/2012/791873

**Published:** 2012-07-18

**Authors:** Mitsushige Sugimoto, Jin Seok Jang, Yashiro Yoshizawa, Satoshi Osawa, Ken Sugimoto, Yoshihiko Sato, Takahisa Furuta

**Affiliations:** ^1^First Department of Medicine, Hamamatsu University School of Medicine, Higashi-ku, Hamamatsu 431-3192, Japan; ^2^Department of Gastroenterology, College of Medicine, Dong-A University, Busan 602-715, Republic of Korea; ^3^Department of Gastroenterology, Seirei General Hospital, Naka-ku, Hamamatsu 430-8558, Japan; ^4^Department of Endoscopic and Photodynamic Medicine, Hamamatsu University School of Medicine, Higashi-ku, Hamamatsu 431-3192, Japan; ^5^Center for Clinical Research, Hamamatsu University School of Medicine, Higashi-ku, Hamamatsu 431-3192, Japan

## Abstract

Endoscopic submucosal dissection (ESD) is a novel endoscopic procedure first developed in the 1990s which enables en bloc resection of gastric neoplastic lesions that are difficult to resect via conventional endoscopic mucosal resection. However, given that ESD increases the risk of intra- and post-ESD delayed bleeding and that platelet aggregation and coagulation in artificial ulcers after ESD strongly depend on intragastric pH, faster and stronger acid inhibition via proton pump inhibitors (PPIs) and histamine 2-receptor antagonists (H_2_RAs) as well as endoscopic hemostasis by thermocoagulation during ESD have been used to prevent ESD-related bleeding. Because PPIs more potently inhibit acid secretion than H_2_RAs, they are often the first-line drugs employed in ESD treatment. However, acid inhibition after the initial infusion of a PPI is weaker in the early phase than that achievable with H_2_RAs; further, PPI effectiveness can vary depending on genetic differences in CYP2C19. Therefore, optimal acid inhibition may require tailored treatment based on CYP2C19 genotype when ESD is performed, with a concomitant infusion of PPI and H_2_RA possibly most effective for patients with the rapid metabolizer CYP2C19 genotype, while PPI alone may be sufficient for those with the intermediate or poor metabolizer genotypes.

## 1. Introduction

Endoscopic submucosal dissection (ESD), an endoscopic procedure that originated from Japan and Korea in the late 1990s and has since spread rapidly to other nations, is now commonly used to treat gastric cancer and adenoma [1]. ESD is performed using electrosurgical knives to make gastrointestinal mucosal incisions and submucosal dissections [2, 3]. Although the procedure requires a high level of endoscopic competence, ESD resection can be performed en bloc, controlling the resected size and shape of tumors and gastric cancer lesions, which are notoriously difficult to resect via conventional endoscopic mucosal resection (EMR). Therefore, ESD allows complete pathological assessment, proving this technique superior to biopsy or EMR for diagnosing gastrointestinal tumors [4]. Further, in most cases, ESD's en bloc approach can be useful in avoiding piecemeal resection, which often leads to a high risk of local recurrence of gastric cancer [5, 6].

Unfortunately, the treatment of relatively large lesions and lesions related to ulcers, ulcer scars, or fibrosis increases the ESD operation time, which subsequently also increases the risk of adverse events such as bleeding and gastrointestinal perforation [7–10]. In fact, the incidence of procedure-related bleeding is higher with ESD than with conventional EMR, meaning the control of bleeding during and after ESD is vital to achieving successful outcomes. In general, ESD-related bleeding is prevented using endoscopic hemostasis and acid inhibition with proton pump inhibitors (PPIs) or histamine 2-receptor antagonists (H_2_RAs). In this papre, we summarize the characteristics of ESD-related bleeding and pharmacotherapy for artificial ulcers after ESD to prevent delayed bleeding in relation to different acid inhibitory drugs and treatment methods.

## 2. Gastric Bleeding as a Complication of ESD

Endoscopic hemostatic methods for countering bleeding from peptic ulcers include various techniques such as local injection of hypertonic saline-epinephrine and ethanol, mechanical hemostasis using endoscopic hemoclips, and thermocoagulation hemostasis. In turn, hemostatic methods in ESD-related bleeding mainly involve thermocoagulation hemostasis using monopolar hemostatic forceps in combination with a water-jet system [11]. This is partly because ESD-related bleeding can lead to intraoperative bleeding and delayed bleeding from exposed vessels at the ulcer base after ESD treatment. Therefore, appropriate management of both is required.

### 2.1. Intraoperative Bleeding

Intraoperative bleeding is inevitable with submucosal local injection and mucosal incision. This is particularly true for ESD when lesions are located in the upper third of the stomach, which involves a relatively higher incidence of intraoperative bleeding given the abundance of vessels [12]. Therefore, identifying these masses of vessels prior to dissection and prophylactic thermocoagulation and the correct layer of the submucosa containing the vessels is important to reduce intraoperative bleeding.

When bleeding does occur during ESD, a clear visual field can be maintained after washing out the blood using the water-jet system, thereby enabling rapid identification of bleeding points.

### 2.2. Hemostasis for Delayed Bleeding

Vessels at the ulcer base often rupture due to physical stimulation by peristalsis or due to chemical stimulation (i.e., bile reflux), such that delayed bleeding after ESD occurs in 0–9% of ESD cases, mostly within 24 h after ESD, in relation to the location of the lesion and ulcer size [5, 13–26]. A combination analysis of 14 reports from Japan (*n* = 6,838) found a delayed bleeding rate of 2.6% (95% confidence interval (CI): 2.3–3.1%) with ESD (Table 1) [5, 13–25]. Higashiyama et al. [21] reported that the risk factors for delayed bleeding after ESD were patients receiving chronic dialysis (*P* = 0.034), operation time ≥75 min (*P* = 0.012), and poor control of bleeding during ESD (*P* = 0.014). Multivariate analysis by Toyokawa et al. [27] showed that age ≥80 years (OR: 2.15, 95% CI: 1.18–3.90) and a long procedure time (OR: 1.01, 95% CI: 1.001–1.007) were associated with a significantly higher risk of delayed bleeding. Further, delayed bleeding after a second-look endoscopy was significantly related with poor control of bleeding during ESD (*P* = 0.04) and operation time ≥75 min (*P* = 0.012) [21]. In a report from Korea, of the five risks factors considered (patient age, lesion size, gross findings, location, and histology of the tumor) for immediate and delayed bleeding associated with endoscopic submucosal dissection of gastric neoplastic, only the tumor histology was statistically significantly associated with bleeding (HR: 6.8, 95% CI: 1.8–25.0, *P* = 0.004) [28]. Moreover, multicenter trial showed that the rates of delayed bleeding differed significantly in relation to location (upper versus lower portion of stomach, 28.6% versus 13.8%, resp.; *P* = 0.003), the size of the tumor (>40 mm versus <20 mm, 28.6% versus 13.7%, resp.; *P* = 0.009), recurrent lesion (29.4% versus 15.1%, resp.; *P* = .024), and macroscopic type (flat versus elevated, 18.8% versus 12.4%, resp.; *P* = .047) [10]. Okada et al. also reported that resected specimen width (≥40 mm) was the only significant factor associated with delayed bleeding on univariate and multivariate analysis [29]. Therefore, one of the major factors for delayed bleeding may be the size of the lesion or resected specimen.

In almost all ESD cases, hemostasis is achieved with urgent endoscopic hemostasis [30]. To prevent delayed bleeding, prophylactic coagulation of the exposed vessels at the base of artificial ulcers is useful. The cause of the delayed bleeding is due more to insufficient prophylactic thermocoagulation than insufficient primary hemostasis during ESD, because in many cases the sites of delayed bleeding and endoscopic hemostasis differ [31]. A Japanese survey of treatment methods for bleeding showed that clipping (32.9%) and coagulation forceps (23.5%) were the most commonly used endoscopic hemostasis methods for countering bleeding from peptic ulcers [32]. In contrast, coagulation forceps (77.8%) were the most commonly used tool to stop bleeding from an artificial ulcer.

### 2.3. Effects of Antiplatelet Drugs for Bleeding

Antiplatelet agents such as low-dose aspirin (LDA) and clopidogrel are used for patients with cardiovascular and cerebrovascular diseases [33]. LDA exerts an antiplatelet effect by decreasing the production of platelet thromboxane B2 via inhibition of cyclooxygenase-1 (COX-1), which often causes gastric mucosal injury [34–38]. We previously reported that esophageal and gastric mucosal damage were respectively observed in 52% and 93% of volunteers using short-term LDA treatment [35, 37, 39], and long-term LDA therapy significantly increases the incidence of gastrointestinal bleeding, a rate is not improved by decreasing the LDA dose or by using an enteric-coated LDA [38].

Recently, Lim et al. [40] reported that the rates of delayed bleeding in patients with the continued use of an anti-platelet drug, the withdrawal of an anti-platelet drug, and the use of non antiplatelet drug were 11.6%, 5.9%, and 5.2%, respectively, while univariate analysis showed that the use of anti-platelet drugs, presence of early gastric cancer and comorbidities, and specimen diameter were related to delayed bleeding. Further, risk of bleeding was high in patients who did not discontinue LDA use (relative risk (RR), 4.5 95* *% CI: 1.1–18.4), while delayed bleeding was more frequent among continuous LDA users (*n* = 9, 21.1* *%) than in those who never used (*n*  =  439, 3.4* *%; *P*  =  0.006) or those with interrupted use for more than 7 days (*n*  =  56, 3.6* *%; *P*   =  0.03) [41]. Concomitant treatment with clopidogrel (RR: 26.7, 95% CI: 7.1–100.5) and increased artificial ulcer size (RR: 1.5, 95% CI: 1.1–* *2.0) were also significantly associated with delayed bleeding. Therefore, to minimize bleeding complications, LDA should be stopped in patients who have low risk for thromboembolic disease.

## 3. Importance of Acid Inhibition in Treatment of Endoscopic Submucosal Dissection

To prevent ESD-related bleeding, pharmacological treatment with PPIs and H_2_RAs as well as endoscopic hemostasis should be considered. Rebleeding up to 72 h after endoscopic treatment is often caused by the dissolution of formed fibrin clots by gastric acid. Because platelet aggregation, coagulation, and fibrinolysis on gastric hemorrhagic ulcers strongly depend on intragastric pH levels [42], ways to neutralize pH levels should be considered [43]. For example, when pH falls below 6.8, platelet aggregation and blood coagulation become abnormal, and when pH falls below pH 5.4, platelet aggregation and plasma coagulation are virtually abolished, while below pH 4.0, fibrin clots are dissolved [42]. Therefore, pH must be elevated to ≥5.5 as quickly as possible and continuously kept above 4.0 (when pepsin is inactivated and fibrinolysis inhibited) [42, 44, 45]. As such, fast and strong acid inhibition in the early postadministration phase is recommended.

## 4. Acid Inhibitory Drugs and Intragastric pH

Currently, PPIs (e.g., omeprazole, lansoprazole, rabeprazole, and esomeprazole) and H_2_RAs (e.g., famotidine, cimetidine, nizatidine, ranitidine, roxatidine, and lafutidine) are widely used as first-line drug therapies for treating not only acid-related diseases but also postendoscopic treatment including ESD and EMR [46].

### 4.1. Pharmacological Characteristics of PPI and H_2_RA

PPIs function by first being absorbed into the small intestine and reaching the gastric parietal cells via systemic circulation, where they then disturb proton pump (H^+^/K^+^-ATPase) activity by irreversibly binding to the pumps, thereby resulting in potent acid inhibition throughout the 24 h postdose period [47, 48]. However, the change in pH after dosing of omeprazole in the early postadministration phase is insufficient, as duration of maintaining pH > 3 for 24 h with omeprazole 20 mg was 13.6%, 35.3%, and 62.8% of the 24 h period for days 1, 2, and 3, respectively [49]. Müller et al. [50] reported that a standard dose of lansoprazole or omeprazole exerted only 30%–60% inhibition on pentagastrin-stimulated acid secretion on days 1 and 2 after drug administration. Based on data regarding acid inhibition by a PPI in the early phase [51, 52], it is generally understood that the first dose inhibits only activated H^+^/K^+^-ATPase present in the canalicular membrane, while actual acid inhibitory effects develop only after the third dose, with maximum acid inhibition achieved on day 5 after drug administration, depending on the degree of activation of H^+^/K^+^-ATPase in the resting phase and on the recovery of disulfide bonds between the PPI and H^+^/K^+^-ATPase.

In contrast, H_2_RAs competitively bind to H_2_-receptors on parietal cells and inhibit acid secretion mediated by histamine [47, 48]. Although PPIs inhibit gastric acid secretion more potently than H_2_RAs overall, PPIs have the disadvantage of exerting relatively weak acid inhibition in the early phase after initial dosing compared with H_2_RAs, which exert their inhibitory effects within a couple of hours of dosing [53]. Indeed, an intravenous infusion of famotidine 20 mg increases pH over 4 h more rapidly than omeprazole 20 mg in *H. pylori-*negative subjects [54, 55].

**Figure 1:**
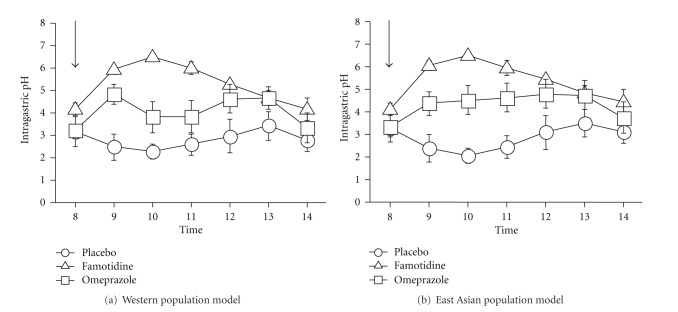
Median 6 h pH-time profiles of intravenous infusions of placebo, famotidine, and omeprazole in a Western population model (CYP2C19 RM (*n* = 7), IM (*n* = 2), and PM (*n* = 1)) (a) and East-Asian population model (CYP2C19 RM (*n* = 3), IM (*n* = 5) and PM (*n* = 2)) (b).

Although acid inhibition by PPI or H_2_RA treatment differs between *H. pylori-*negative and *H. pylori-*positive subjects, few reports have discussed changes in pH during the early phase or the increasing pH value in *H. pylori-*positive patients [49, 53]. We previously reported the presence of differences between PPI and H_2_RA with regard to their potency and time to acid inhibition in healthy *H. pylori-*positive subjects. In the median 6 h pH-time profiles of intravenous infusions of famotidine and omeprazole in Western (CYP2C19 RM (*n* = 7), IM (*n* = 2) and PM (*n* = 1)) and East-Asian population models (CYP2C19 RM (*n* = 3), IM (*n* = 5) and PM (*n* = 2)), the median pH with famotidine was higher than that with omeprazole (Figures 1(a) and 1(b)), and the acid inhibition early post-administration with famotidine 20 mg was more potent than that with omeprazole 20 mg [56]. Therefore, for artificial ulcers within 24 h after ESD, treatment with H_2_RA drugs may be more appropriate than that using PPIs [57].

### 4.2. CYP2C19 and Acid Inhibitory Drugs

**Figure 2:**
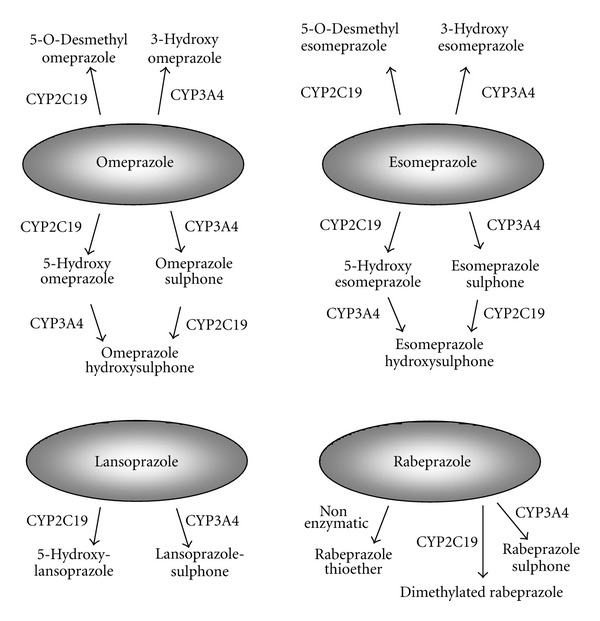
Metabolic pathways of esomeprazole, omeprazole, lansoprazole, and rabeprazole in relation to cytochrome P450 isoenzymes CYP2C19 and CYP3A4. Weight of arrows indicates the relative contribution of the different enzyme pathways.

**Figure 3:**
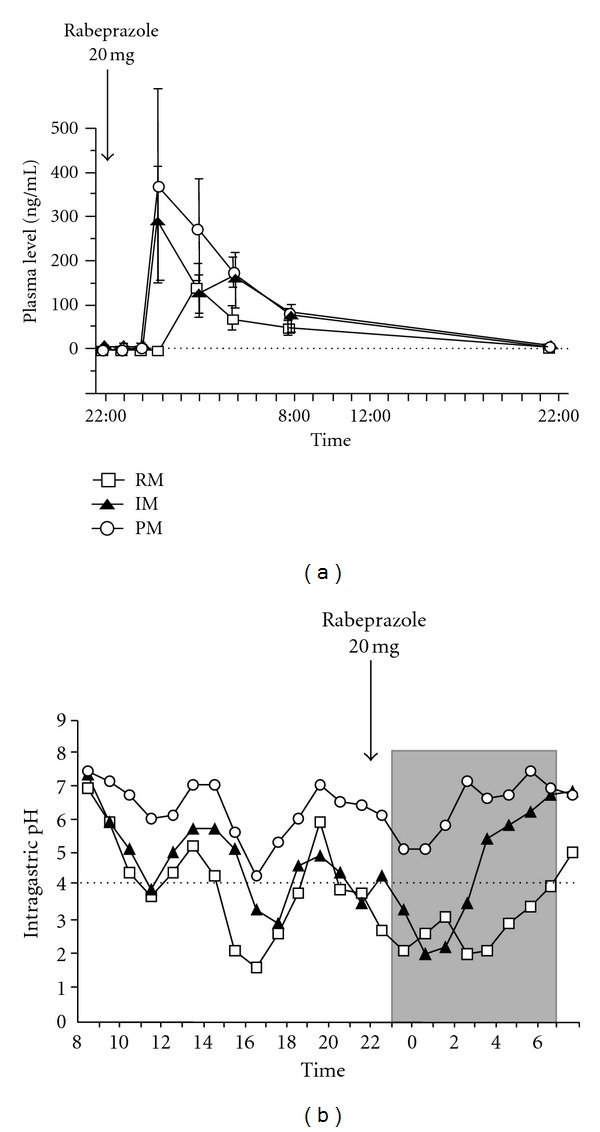
Plasma rabeprazole levels (a) and 24 h pH profiles (b) after rabeprazole 20 mg od treatment as a function of the CYP2C19 genotype group [58]. Abbreviations: RM, rapid extensive metabolizer; IM, intermediate extensive metabolizer; PM, poor metabolizer.

PPIs undergo extensive hepatic metabolism by the CYP system, particularly by CYP2C19 (Figure 2) [59]. As such, pharmacokinetics (i.e., the peak plasma concentration (C_max_) and area under the plasma concentration (AUC) of a PPI) and pharmacodynamics of PPIs (i.e., intragastric pH) differ significantly by CYP2C19 genotype [58, 60–62]. Although more than 20 variant alleles of CYP2C19 have been discovered, the majority of individuals in Japanese and Korean populations can be classified into three genotypes: rapid (RM), intermediate (IM), and poor metabolizers (PM), based on the CYP2C19 wild-type (CYP2C19 *1) gene and two mutated alleles (CYP2C19*2 (*2) in exon 5 and CYP2C19*3 (*3) in exon 4) [59, 63, 64]. In CYP2C19 PMs, plasma PPI concentrations are markedly increased, while acid inhibition by PPIs is enhanced in comparison with that in RMs and IMs, with the acid inhibition attained in RMs sometimes being insufficient for positive outcomes (Figures 3(a) and 3(b)) [58, 61, 62, 65–67]. Therefore, it may be important to consider the interethnic differences in frequency of CYP2C19 PM when treating with a PPI, with rates of 2.5–3.5% in Caucasians, 13.4–19.8% in Chinese, 12.6% in Koreans, and 18.0–22.5% in Japanese [64, 68, 69].

A recent study reported that the AUC of PPIs in subjects with the CYP2C19 *17/*17 genotype, an ultra-rapid metabolizer genotype of CYP2C19, was up to 40% lower than that of the CYP2C19 *1/*1 genotype [70]. The frequency of the *17 allele also appears to vary with ethnicity, present in 27.2% of Poles and 18% of Ethiopians and Swedes while in only 4% of Chinese and less than 2% of Japanese [70–72]. East Asians clearly exert lower CYP2C19 activity due to the higher frequency of CYP2C19 PMs as well as a lower frequency of ultrarapid EMs (*17 carrier) [70]. These findings contrast sharply with those achieved with H_2_RAs, whose metabolism is not affected by CYP2C19 genotype [62, 73]. H_2_RAs are mainly excreted in their unchanged form from the urine without any hepatic metabolism by CYP enzymes [73]. Therefore, the pharmacokinetics and pharmacodynamics of H_2_RAs are not influenced by the CYP2C19 genotype status [62, 73].

**Figure 4:**
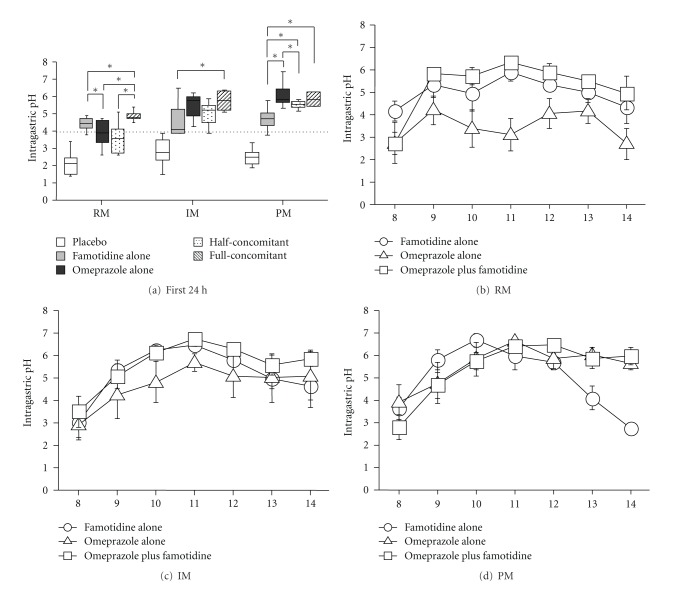
Median 12 h pH level in different intravenous infusion regimens (placebo, famotidine 20 mg, omeprazole 20 mg, famotidine 10 mg plus omeprazole 10 mg (half-concomitant), famotidine 20 mg plus omeprazole 20 mg (full-concomitant)) in CYP2C19 RM, IM, and PM genotype groups (a). Median 6-h pH-time profiles of intravenous infusions of famotidine alone, omeprazole alone, and a concomitant of famotidine and omeprazole in the RM (b), IM (c), and PM genotype groups (d). *P < 0.05 using the Wilcoxon's signed rank test, when significant differences were obtained by the Friedmann's test.

In our previous report in *H. pylori-*positive subjects, the pH following infusion of famotidine 20 mg bid (4.4 (3.8–4.9)) in RMs in the first 24 h was higher than that achieved with infusion of omeprazole 20 mg bid (3.9 (2.6–4.7)), with more effective acid inhibition during the early phase in CYP2C19 RMs as well (Table 2 and Figure 4(a)) [56]. In contrast, in I PMs, the pH during the first 24 h period with omeprazole was significantly higher than that attained by famotidine (Table 2 and Figure 4(a)). Therefore, CYP2C19 genotyping appears to be a useful tool for determining optimal treatment to prevent bleeding from artificial ulcers and delayed bleeding from artificial ulcers within 24 h after ESD, as the onset of gastric acid secretion inhibition by H_2_RA drugs is more rapid than that of PPIs, suggesting that H_2_RA may be more effective, particularly in CYP2C19 RMs, than PPI [57].

### 4.3. Tailored Treatment for Potent Acid Inhibition throughout 24 h

In RMs, PPIs are rapidly eliminated from the systemic circulation, resulting in insufficient acid inhibition such that newly generated or activated in gastric parietal cells after the rapid elimination of PPI can secrete gastric acid. However, with multiple doses of a PPI, plasma PPI levels can be sustained throughout the 24 h period and can continue to inactivate H^+^, K^+^-ATPase consistently for 24 h, resulting in sufficient acid inhibition during treatment. When rabeprazole 40 mg od or 20 mg bid is administered to RMs, plasma levels are often below detectable levels [58]. However, with rabeprazole 20 mg bid in IMs and 10 mg qid in RMs, plasma levels are sustained above 10 ng/mL throughout the 24 h period, and sufficient acid suppression is achieved [58].

## 5. Anti-Acid Drugs for Artificial Ulcers

### 5.1. Bleeding

Delayed bleeding occurs mostly within 24 h after ESD. Further, gastric pH affects the efficiency of blood coagulation and platelet aggregation at the bleeding site. When comparing PPIs and H_2_RAs for the prevention of acute phase delayed bleeding from artificial ulcers within 24 h after ESD, H_2_RAs, whose onset of acid inhibition is more rapid, may be more effective when the drug is dosed on the day of ESD [57]. However, Uedo et al. [74] reported that the pH was significantly higher in patients administered PPIs than in those receiving H_2_RAs on the day before ESD, while we found that PPI bid infusion effectively increased the pH more than H_2_RAs infusion did on the second day, even in the CYP2C19 RM group (Table 2) [56].

In contrast, in the chronic phase, more than three days after ESD, PPI exerts stronger acid inhibition, even in the CYP2C19 RM group [62]. Uedo et al. [74] reported that delayed bleeding after ESD occurred in 6.1% of patients treated with rabeprazole and 17.2% treated with cimetidine, and that PPI therapy more effectively prevented delayed bleeding from artificial ulcers than did H_2_RA. Further, multivariate analysis showed that PPI treatment for 8 weeks was an independent factor in reducing the rate of delayed bleeding. Also the meta-analysis of 6 full-text studies that included a total of 522 patients showed a significantly lower delayed bleeding rate in patients that received PPIs than in those receiving H_2_RA (OR: 0.49, 95% CI: 0.25–0.95) [75].

However, Imaeda et al. [23] recently reported no significant difference between the PPI lansoprazole or the H_2_RA roxatidine in preventing delayed bleeding after ESD over 8 weeks' treatment (3.2% versus 4.9%, resp.). Similarly, Yamaguchi et al. reported no significant difference between famotidine and omeprazole recipients in delayed bleeding (18% versus 14%, resp.) [57].

With regard to when physicians should begin treatment with a PPI, Ono et al. [76] reported that although pH in a postoperative group that received omeprazole after ESD was lower than that among patients administered omeprazole from the day before ESD, no significant difference was noted in major and minor delayed bleeding ratios between the two (7.7% versus 7.4%, resp.).

### 5.2. Healing

Multivariate analysis has shown that initial artificial ulcer size and duration of PPI treatment after ESD are correlated with both the marginal and basal healing rates [77]. Also, the marginal healing rate in the antrum is higher than that of ulcer lesions in other areas of the body. However, *H. pylori *infection and the extent of gastric atrophy do not affect ulcer healing when concomitant treatment of PPI and gastric mucosa protective agent for eight weeks after ESD is performed [78]. Multivariate logistic regression of retrospective data showed that the treatment periods of PPI and ulcer size are associated with ulcer healing, with a duration of PPI treatment of <8 weeks being required to heal post-ESD ulcers ≥40 mm [79]. The same study found in a prospective validation that the rate of complete healing of artificial ulcers in an 8-week PPI group was significantly higher than that of a 4-week group at an 8-week followup (83.3 versus 42.6%, resp.; *P* < 0.01) [79]. Consistent with this, Kakushima et al. [14] reported that four weeks of PPI administration was not sufficient; instead eight weeks was required to obtain satisfactory results for larger ulcers. Therefore, the optimal duration of PPI treatment to treat ESD-induced ulcers should be eight weeks.

The concomitant treatment of a PPI and gastric mucosa protective agent, rebamipide, has a significantly higher rate of basal healing on large-sized artificial ulcers than that in PPI alone (*P* = 0.015) [77]. In a randomized prospective controlled study of 290 patients (309 lesions), the ulcer healing rates at 4 weeks after ESD in the concomitant treatment group (94.9%) were significantly higher than those in the PPI alone group (89.9%; *P* < 0.0001) [80]. Additionally, this combination therapy was found to be an independent predictive factor for a relatively high healing rate (OR 5.6; 95% CI, 2.6–11.9; *P* = 0.014). Fujiwara et al. [81] also reported that among patients with severe atrophic gastritis (the O-3 type according to Kimura-Takemoto classification), the healing-to-scarring stage occurred in 30.0% of patients in the PPI alone group and in 92.9% in the PPI and rebamipide (OR: 30.3, 95% CI: 2.6–348.9) combination group after 8 weeks of treatment. Overall, treatment with a PPI plus gastric mucosa protective agents led to improved healing for patients with ESD-derived artificial ulcers, particularly among those with severe atrophic gastritis.

Comparison analysis demonstrates the usefulness of PPIs and H_2_RAs in healing artificial ulcers after ESD, while meta-analysis shows significantly higher ulcer healing rates with PPIs than with H_2_RAs [82, 83]. For example, Ye et al. [84] reported that active ulcers remained at a higher incidence after four weeks of H_2_RA treatment than of PPI treatment in artificial ulcers. However, a recent report showed similar healing rates and the rates of decrease in ulcer size in Japanese patients treated with H_2_RAs and PPIs (healing rate: 93.5% (58/62) in lansoprazole and 93.4% (57/61) in roxatidine [23]; rate of decrease in ulcer size: 99.7% in rabeprazole 10 mg and 99.8% in roxatidine 150 mg [85]) after ESD for 8 weeks. Further, there had been a few studies comparing with full dose and half dose of PPI for treating artificial ulcers after ESD. Kawano et al. [22] reported that after treating treatment with a standard dose of a PPI during the first week, the rates of ulcers healing and scores on the gastrointestinal symptom rating scale were similar in patients receiving standard and half doses of lansoprazole for 8 weeks.

### 5.3. Cost-Effectiveness

Yamaguchi et al. [57] reported that although delayed bleeding rates show no significant difference between treatments with famotidine or omeprazole, their costs ($130.25/8-weeks treatment for famotidine versus $222.28/8-weeks treatment for omeprazole) suggest famotidine is the better option. Imaeda et al. [23] made a similar argument for the use of H_2_RAs, as the cost of PPI lansoprazole is $165.15 while that of H_2_RA roxatidine is $73.01 for 8-week treatment. Similarly, half doses of PPI are economically preferred to standard doses ($91.58 versus $146.25) [22].

## 6. Optimal Treatment

The optimal infusion dose of acid inhibitory drugs and the optimal treatment methods for the treatment of artificial ulcers after ESD have not yet been established. Recently, a concomitant dosage regimen of a PPI with an H_2_RA has been reported to inhibit acid secretion more effectively than an increasing dosage regimen of a PPI or H_2_RA alone [67, 86–88]. However, whether or not the pharmacodynamic effects of concomitant intravenous infusions of a PPI and an H_2_RA on acid inhibition in relation to different CYP2C19 genotypes are beneficial in patients treated with ESD remains obscure. We previously reported that in CYP2C19 RMs, the median pH with concomitant intravenous infusions (4.8 (4.5–5.4)) was higher than that with famotidine (4.4 (3.8–4.9), *P* = 0.043) or omeprazole (3.9 (2.6–4.7), *P* = 0.043) alone (Table 2 and Figure 4(b)) [56]. In contrast, median pH in IMs and PMs was fairly similar between the omeprazole and concomitant regimens but greater than that attained with famotidine (Table 2 and Figures 4(b) and 4(c)). In the concomitant infusion, the median pH with RMs (4.8 (4.5–5.4)) was significantly lower than that with IMs (5.8 (5.1–6.4), *P* = 0.028) or PMs (5.8 (5.4–6.2), *P* = 0.016). Because the major stimulator of nocturnal acid secretion is histamine, an H_2_RA may effectively inhibit such secretion [62, 89], meaning that concomitant treatment of a PPI with an H_2_RA may overcome any weaknesses in PPI inhibition of nighttime acid inhibition. A concomitant intravenous infusion regimen of omeprazole 20 mg and famotidine 20 mg for two days showed a significantly faster onset of raising pH and significantly stronger inhibition of gastric acid secretion, particularly in CYP2C19 RMs, than omeprazole 20 mg alone or famotidine 20 mg alone, although sufficient acid inhibition was able to be achieved in IMs and PMs with omeprazole treatment alone. Therefore, concomitant treatment with an H_2_RA and a PPI can compensate for any disadvantages of a PPI alone during the early post-administration phase in the RM genotype group. We are therefore tempted to recommend this test independent of patient ethnicity when deciding optimal treatment.

## 7. Summary

In conclusion, CYP2C19 genotyping appears to be useful in determining optimal treatment to prevent bleeding from artificial ulcers. If CYP2C19 genotype is clear before ESD, an optimal intravenous infusion regimen consisting of a PPI and an H_2_RA can be selected based on the patient's pharmacogenetic and pharmacogenomic status. The following intravenous infusion regimens are recommended for patients who require intensive gastric acid control during the early post-administration phase: omeprazole 20 mg twice daily for CYP2C19 PM and IM patients and concomitant infusion of omeprazole 20 mg and famotidine 20 mg twice daily for CYP2C19 RM patients. It should be noted that whether or not outcomes such as bleeding rate and healing rate are associated with this treatment remains unclear.

## Figures and Tables

**Table 1:** Delayed bleeding rate of endoscopic submucosal dissection for gastric cancer and gastric adenoma in Japanese patients.

Author	Year	Lesions	Delayed bleeding (%)	Resection rate (%)
Imagawa et al. [13]	2006	196	0	93
Kakushima et al. [14]	2006	383	3.4	91
Oka et al. [5]	2006	195	6.2	83
Onozato et al. [15]	2006	171	7.6	94
Hirasaki et al. [16]	2007	112	7.1	96
Ono et al. [17]	2008	161	8.7	99
Isomoto et al. [18]	2009	510	1.8	95
Hoteya et al. [19]	2009	572	4.9	95
Tsuji et al. [20]	2010	398	5.8	NA
Higashiyama et al. [21]	2011	924	3.0	NA
Kawano et al. [22]	2011	91	2.2	97.8
Imaeda et al. [23]	2011	123	4.1	97.7
Akasaka et al. [24]	2011	1188	3.1	95
Goto et al. [25]	2012	1814	5.5	NA

Total		6838	2.6 (95% CI: 2.3–3.1)	

NA: not analyzed; CI: confidence interval.

**Table 2:** Median intragastric pH during the first 24 and 48 h with different intravenous infusion regimens as a function of CYP2C19 genotype status.

Regimen	Periods	RM	IM	PM
Placebo	First 24 h	2.2 (1.3–3.6)	2.8 (1.8–3.5)	2.4 (1.5–2.9)
	First 48 h	2.1 (1.5–3.4)	2.8 (1.5–3.9)	2.4 (1.9–3.3)
Famotidine	First 24 h	4.4 (3.8–4.9)	4.1 (3.9–6.5)	4.7 (3.7–5.7)
	First 48 h	4.2 (3.5–4.6)	4.0 (3.8–6.1)	4.3 (3.6–4.9)
Omeprazole	First 24 h	3.9 (2.6–4.7)	5.8 (4.3–6.3)^∗^	6.1 (5.3–7.4)^∗^
	First 48 h	4.8 (3.2–5.3)	6.0 (5.4–6.5)^∗^	6.1 (5.7–7.5)^∗^
Concomitant	First 24 h	4.8 (4.5–5.4)	5.8 (5.1–6.4)^∗^	5.8 (5.4–6.2)^∗^
	First 48 h	5.3 (4.7–5.4)	5.7 (5.5–6.4)^∗^	5.9 (5.5–6.2)^∗^

^∗^: *P* < 0.05 (versus RM).

## Abbreviations

CYP2C19: S-mephenytoin 4-hydroxylase
EGRD: Gastroesophageal reflux disease
EMR: Endoscopic mucosal resection
ESD: Endoscopic submucosal dissection
H_2_RA: Histamine 2-receptor antagonist
IM: Intermediate metabolizer
NERD: Nonerosive reflux disease
PPI: Proton pump inhibitor
PM: Poor metabolizer
RM: Rapid metabolizer.

## References

[1] Fujishiro M (2006). Endoscopic submucosal dissection for stomach neoplasms. *World Journal of Gastroenterology*.

[2] Ono H, Kondo H, Gotoda T (2001). Endoscopic mucosal resection for treatment of early gastric cancer. *Gut*.

[3] Gotoda T (2005). A large endoscopic resection by endoscopic submucosal dissection procedure for early gastric cancer. *Clinical Gastroenterology and Hepatology*.

[4] Hull MJ, Mino-Kenudson M, Nishioka NS (2006). Endoscopic mucosal resection: an improved diagnostic procedure for early gastroesophageal epithelial neoplasms. *The American Journal of Surgical Pathology*.

[5] Oka S, Tanaka S, Kaneko I (2006). Advantage of endoscopic submucosal dissection compared with EMR for early gastric cancer. *Gastrointestinal Endoscopy*.

[6] Watanabe K, Ogata S, Kawazoe S (2006). Clinical outcomes of EMR for gastric tumors: historical pilot evaluation between endoscopic submucosal dissection and conventional mucosal resection. *Gastrointestinal Endoscopy*.

[7] Fujishiro M, Yahagi N, Kakushima N (2006). Successful nonsurgical management of perforation complicating endoscopic submucosal dissection of gastrointestinal epithelial neoplasms. *Endoscopy*.

[8] Tanaka M, Ono H, Hasuike N, Takizawa K (2008). Endoscopic submucosal dissection of early gastric cancer. *Digestion*.

[9] Cao Y, Liao C, Tan A, Gao Y, Mo Z, Gao F (2009). Meta-analysis of endoscopic submucosal dissection versus endoscopic mucosal resection for tumors of the gastrointestinal tract. *Endoscopy*.

[10] Chung IK, Lee JH, Lee SH (2009). Therapeutic outcomes in 1000 cases of endoscopic submucosal dissection for early gastric neoplasms: Korean ESD Study Group multicenter study. *Gastrointestinal Endoscopy*.

[11] Enomoto S, Yahagi N, Fujishiro M (2007). Novel endoscopic hemostasis technique for use during endoscopic submucosal dissection. *Endoscopy*.

[12] Hirao M, Masuda K, Asanuma T (1988). Endoscopic resection of early gastric cancer and other tumors with local injection of hypertonic saline-epinephrine. *Gastrointestinal Endoscopy*.

[13] Imagawa A, Okada H, Kawahara Y (2006). Endoscopic submucosal dissection for early gastric cancer: results and degrees of technical difficulty as well as success. *Endoscopy*.

[14] Kakushima N, Fujishiro M, Kodashima S, Muraki Y, Tateishi A, Omata M (2006). A learning curve for endoscopic submucosal dissection of gastric epithelial neoplasms. *Endoscopy*.

[15] Onozato Y, Ishihara H, Iizuka H (2006). Endoscopic submucosal dissection for early gastric cancers and large flat adenomas. *Endoscopy*.

[16] Hirasaki S, Kanzaki H, Matsubara M (2007). Treatment of over 20 mm gastric cancer by endoscopic submucosal dissection using an insulation-tipped diathermic knife. *World Journal of Gastroenterology*.

[17] Ono H, Hasuike N, Inui T (2008). Usefulness of a novel electrosurgical knife, the insulation-tipped diathermic knife-2, for endoscopic submucosal dissection of early gastric cancer. *Gastric Cancer*.

[18] Isomoto H, Shikuwa S, Yamaguchi N (2009). Endoscopic submucosal dissection for early gastric cancer: a large-scale feasibility study. *Gut*.

[19] Hoteya S, Iizuka T, Kikuchi D, Yahagi N (2009). Benefits of endoscopic submucosal dissection according to size and location of gastric neoplasm, compared with conventional mucosal resection. *Journal of Gastroenterology and Hepatology*.

[20] Tsuji Y, Ohata K, Ito T (2010). Risk factors for bleeding after endoscopic submucosal dissection for gastric lesions. *World Journal of Gastroenterology*.

[21] Higashiyama M, Oka S, Tanaka S (2011). Risk factors for bleeding after endoscopic submucosal dissection of gastric epithelial neoplasm. *Digestive Endoscopy*.

[22] Kawano S, Okada H, Kawahara Y (2011). Proton pump inhibitor dose-related healing rate of artificial ulcers after endoscopic submucosal dissection: a prospective randomized controlled trial. *Digestion*.

[23] Imaeda H, Hosoe N, Suzuki H (2011). Effect of lansoprazole versus roxatidine on prevention of bleeding and promotion of ulcer healing after endoscopic submucosal dissection for superficial gastric neoplasia. *Journal of Gastroenterology*.

[24] Akasaka T, Nishida T, Tsutsui S (2011). Short-term outcomes of endoscopic submucosal dissection (ESD) for early gastric neoplasm: multicenter survey by osaka university ESD study group. *Digestive Endoscopy*.

[25] Goto O, Fujishiro M, Oda I (2012). A multicenter survey of the management after gastric endoscopic submucosal dissection related to postoperative bleeding. *Digestive Diseases and Sciences*.

[26] Gotoda T, Yamamoto H, Soetikno RM (2006). Endoscopic submucosal dissection of early gastric cancer. *Journal of Gastroenterology*.

[27] Toyokawa T, Inaba T, Omote S (2012). Risk factors for perforation and delayed bleeding associated with endoscopic submucosal dissection for early gastric neoplasms, analysis of 1123 lesions. *Journal of Gastroenterology and Hepatology*.

[28] Jang JS, Choi SR, Graham DY (2009). Risk factors for immediate and delayed bleeding associated with endoscopic submucosal dissection of gastric neoplastic lesions. *Scandinavian Journal of Gastroenterology*.

[29] Okada K, Yamamoto Y, Kasuga A (2011). Risk factors for delayed bleeding after endoscopic submucosal dissection for gastric neoplasm. *Surgical Endoscopy and Other Interventional Techniques*.

[30] Messmann H, Probst A (2009). Management of endoscopic submucosal dissection complications. *Endoscopy*.

[31] Takizawa K, Oda I, Gotoda T (2008). Routine coagulation of visible vessels may prevent delayed bleeding after endoscopic submucosal dissection—an analysis of risk factors. *Endoscopy*.

[32] Fujishiro M, Abe N, Endo M (2010). Current managements and outcomes of peptic and artificial ulcer bleeding in Japan. *Digestive Endoscopy*.

[33] Awtry EH, Loscalzo J (2000). Aspirin. *Circulation*.

[34] Taha AS, Angerson WJ, Knill-Jones RP, Blatchford O (2005). Upper gastrointestinal haemorrhage associated with low-dose aspirin and anti-thrombotic drugs—a 6-year analysis and comparison with non-steroidal anti-inflammatory drugs. *Alimentary Pharmacology and Therapeutics*.

[35] Nishino M, Sugimoto M, Kodaira C (2010). Relationship between low-dose aspirin-induced gastric mucosal injury and intragastric pH in healthy volunteers. *Digestive Diseases and Sciences*.

[36] Sugimoto M, Nishino M, Kodaira C (2011). Impact of acid inhibition on esophageal mucosal injury induced by low-dose aspirin. *Digestion*.

[37] Sugimoto M, Nishino M, Kodaira C (2010). Esophageal mucosal injury with low-dose aspirin and its prevention by rabeprazole. *Journal of Clinical Pharmacology*.

[38] Derry S, Loke YK (2000). Risk of gastrointestinal haemorrhage with long term use of aspirin: meta-analysis. *British Medical Journal*.

[39] Nishino M, Sugimoto M, Kodaira C (2011). Preventive effects of lansoprazole and famotidine on gastric mucosal injury induced by low-dose aspirin in Helicobacter pylori-negative healthy volunteers. *Journal of Clinical Pharmacology*.

[40] Lim JH, Kim SG, Kim JW (2012). Do antiplatelets increase the risk of bleeding after endoscopic submucosal dissection of gastric neoplasms?. *Gastrointestinal Endoscopy*.

[41] Cho SJ, Choi IJ, Kim CG (2012). Aspirin use and bleeding risk after endoscopic submucosal dissection in patients with gastric neoplasms. *Endoscopy*.

[42] Green FW, Kaplan MM, Curtis LE, Levine PH (1978). Effect of acid and pepsin on blood coagulation and platelet aggregation: a possible contributor to prolonged gastroduodenal mucosal hemorrhage. *Gastroenterology*.

[43] Barer D, Ogilvie A, Henry D (1983). Cimetidine and tranexamic acid in the treatment of acute upper-gastrointestinal-tract bleeding. *The New England Journal of Medicine*.

[44] Labenz J, Peitz U, Leusing C, Tillenburg B, Blum AL, Börsch G (1997). Efficacy of primed infusions with high dose ranitidine and omeprazole to maintain high intragastric pH in patients with peptic ulcer bleeding: a prospective randomised controlled study. *Gut*.

[45] Andersen J, Strom M, Naesdal J, Leire K, Walan A (1990). Intravenous omeprazole: effect of a loading dose on 24-h intragastric pH. *Alimentary Pharmacology and Therapeutics*.

[46] Klinkenberg-Knol EC, Festen HPM, Jansen JB (1994). Long-term treatment with omeprazole for refractory reflux esophagitis: efficacy and safety. *Annals of Internal Medicine*.

[47] Hixson LJ, Kelley CL, Jones WN, Tuohy CD (1992). Current trends in the pharmacotherapy for peptic ulcer disease. *Archives of Internal Medicine*.

[48] Sachs G, Shin JM, Briving C, Wallmark B, Hersey S (1995). The pharmacology of the gastric acid pump: the H^+^, K^+^ ATPase. *Annual Review of Pharmacology and Toxicology*.

[49] Saitoh T, Fukushima Y, Otsuka H (2002). Effects of rabeprazole, lansoprazole and omeprazole on intragastric pH in CYP2C19 extensive metabolizers. *Alimentary Pharmacology and Therapeutics*.

[50] Müller P, Göksu MA, Fuchs W, Schlüter F, Simon B (2000). Initial potency of lansoprazole and omeprazole tablets on pentagastrin-stimulated gastric acid secretion—a placebo-controlled study in healthy volunteers. *Alimentary Pharmacology and Therapeutics*.

[51] Howden CW, Forrest JAH, Reid JL (1984). Effects of single and repeated doses of omeprazole on gastric acid and pepsin secretion in man. *Gut*.

[52] Jansen JBMJ, Lundborg P, Baak LC (1988). Effect of single and repeated intravenous doses of omeprazole on pentagastrin stimulated gastric acid secretion and pharmacokinetics in man. *Gut*.

[53] Abe Y, Inamori M, Togawa JI (2004). The comparative effects of single intravenous doses of omeprazole and famotidine on intragastric pH. *Journal of Gastroenterology*.

[54] Khoury RM, Katz PO, Castell DO (1999). Post-prandial ranitidine is superior to post-prandial omeprazole in control of gastric acidity in healthy volunteers. *Alimentary Pharmacology and Therapeutics*.

[55] Arnestad JS, Kleveland PM, Waldum HL (1997). In single doses ranitidine effervescent is more effective than lansoprazole in decreasing gastric acidity. *Alimentary Pharmacology and Therapeutics*.

[56] Sugimoto M, Furuta T, Shirai N, Ikuma M, Hishida A, Ishizaki T (2006). Initial 48-hour acid inhibition by intravenous infusion of omeprazole, famotidine, or both in relation to cytochrome P450 2C19 genotype status. *Clinical Pharmacology & Therapeutics*.

[57] Yamaguchi Y, Katsumi N, Tauchi M (2005). A prospective randomized trial of either famotidine or omeprazole for the prevention of bleeding after endoscopic mucosal resection and the healing of endoscopic mucosal resection-induced ulceration. *Alimentary Pharmacology and Therapeutics, Supplement*.

[58] Sugimoto M, Furuta T, Shirai N (2004). Different dosage regimens of rabeprazole for nocturnal gastric acid inhibition in relation to cytochrome P450 2C19 genotype status. *Clinical Pharmacology & Therapeutics*.

[59] Ishizaki T, Horai Y (1999). Review article: cytochrome P450 and the metabolism of proton pump inhibitors—emphasis on rabeprazole. *Alimentary Pharmacology and Therapeutics, Supplement*.

[60] Horai Y, Kimura M, Furuie H (2001). Pharmacodynamic effects and kinetic disposition of rabeprazole in relation to CYP2C19 genotypes. *Alimentary Pharmacology and Therapeutics*.

[61] Shirai N, Furuta T, Moriyama Y (2001). Effects of CYP2C19 genotypic differences in the metabolism of omeprazole and rabeprazole on intragastric pH. *Alimentary Pharmacology and Therapeutics*.

[62] Shirai N, Furuta T, Xiao F (2002). Comparison of lansoprazole and famotidine for gastric acid inhibition during the daytime and night-time in different CYP2C19 genotype groups. *Alimentary Pharmacology and Therapeutics*.

[63] Chang M, Dahl ML, Tybring G, Götharson E, Bertilsson L (1995). Use of omeprazole as a probe drug for CYP2C19 phenotype in swedish caucasians: comparison with S-mephenytoin hydroxylation phenotype and CYP2C19 genotype. *Pharmacogenetics*.

[64] Kubota T, Chiba K, Ishizaki T (1996). Genotyping of S-mephenytoin 4’-hydroxylation in an extended Japanese population. *Clinical Pharmacology & Therapeutics*.

[65] Furuta T, Ohashi K, Kosuge K (1999). CYP2C19 genotype status and effect of omeprazole on intragastric pH in humans. *Clinical Pharmacology & Therapeutics*.

[66] Furuta T, Shirai N, Takashima M (2001). Effects of genotypic differences in CYP2C19 status on cure rates for Helicobacter pylori infection by dual therapy with rabeprazole plus amoxicillin. *Pharmacogenetics*.

[67] Sugimoto M, Furuta T, Shirai N (2005). Comparison of an increased dosage regimen of rabeprazole versus a concomitant dosage regimen of famotidine with rabeprazole for nocturnal gastric acid inhibition in relation to cytochrome P450 2C19 genotypes. *Clinical Pharmacology & Therapeutics*.

[68] Ishizaki T, Sohn DR, Kobayashi K (1994). Interethnic differences in omeprazole metabolism in the two S-mephenytoin hydroxylation phenotypes studied in Caucasians and Orientals. *Therapeutic Drug Monitoring*.

[69] de Morais SM, Goldstein JA, Xie HG (1995). Genetic analysis of the S-mephenytoin polymorphism in a Chinese population. *Clinical Pharmacology & Therapeutics*.

[70] Sim SC, Risinger C, Dahl ML (2006). A common novel CYP2C19 gene variant causes ultrarapid drug metabolism relevant for the drug response to proton pump inhibitors and antidepressants. *Clinical Pharmacology & Therapeutics*.

[71] Kurzawski M, Gawrońska-Szklarz B, Wrześniewska J, Siuda A, Starzyńska T, Droździk M (2006). Effect of CYP2C19∗17 gene variant on Helicobacter pylori eradication in peptic ulcer patients. *European Journal of Clinical Pharmacology*.

[72] Sugimoto K, Uno T, Yamazaki H, Tateishi T (2008). Limited frequency of the CYP2C19∗17 allele and its minor role in a Japanese population. *British Journal of Clinical Pharmacology*.

[73] Gladziwa U, Klotz U (1994). Pharmacokinetic optimisation of the treatment of peptic ulcer in patients with renal failure. *Clinical Pharmacokinetics*.

[74] Uedo N, Takeuchi Y, Yamada T (2007). Effect of a proton pump inhibitor or an H2-receptor antagonist on prevention of bleeding from ulcer after endoscopic submucosal dissection of early gastric cancer: a prospective randomized controlled trial. *The American Journal of Gastroenterology*.

[75] Yang Z, Wu Q, Liu Z (2011). Proton pump inhibitors versus histamine-2-receptor antagonists for the management of iatrogenic gastric ulcer after endoscopic mucosal resection or endoscopic submucosal dissection: a meta-analysis of randomized trials. *Digestion*.

[76] Ono S, Kato M, Ono Y (2009). Effects of preoperative administration of omeprazole on bleeding after endoscopic submucosal dissection: a prospective randomized controlled trial. *Endoscopy*.

[77] Kobayashi M, Takeuchi M, Hashimoto S (2012). Contributing factors to gastric ulcer healing after endoscopic submucosal dissection including the promoting effect of rebamipide. *Digestive Diseases and Sciences*.

[78] Kakushima N, Fujishiro M, Yahagi N, Kodashima S, Nakamura M, Omata M (2006). Helicobacter pylori status and the extent of gastric atrophy do not affect ulcer healing after endoscopic submucosal dissection. *Journal of Gastroenterology and Hepatology*.

[79] Lee SH, Lee CK, Chung IK (2012). Optimal duration of proton pump inhibitor in the treatment of endoscopic submucosal dissection-induced ulcers: a retrospective analysis and prospective validation study. *Digestive Diseases and Sciences*.

[80] Shin WG, Kim SJ, Choi MH (2012). Can rebamipide and proton pump inhibitor combination therapy promote the healing of endoscopic submucosal dissection-induced ulcers? A randomized, prospective, multicenter study. *Gastrointestinal Endoscopy*.

[81] Fujiwara S, Morita Y, Toyonaga T (2011). A randomized controlled trial of rebamipide plus rabeprazole for the healing of artificial ulcers after endoscopic submucosal dissection. *Journal of Gastroenterology*.

[82] Di Mario F, Battaglia G, Leandro G, Grasso G, Vianello F, Vigneri S (1996). Short-term treatment of gastric ulcer: a meta-analytical evaluation of blind trials. *Digestive Diseases and Sciences*.

[83] Tunis SR, Sheinhait IA, Schmid CH, Bishop DJ, Ross SD (1997). Lansoprazole compared with histamine2-receptor antagonists in healing gastric ulcers: a meta-analysis. *Clinical Therapeutics*.

[84] Ye BD, Cheon JH, Choi KD (2006). Omeprazole may be superior to famotidine in the management of iatrogenic ulcer after endoscopic mucosal resection: a prospective randomized controlled trial. *Alimentary Pharmacology and Therapeutics*.

[85] Takeuchi N, Umegaki E, Takeuchi T (2011). Gastric ulcer healing after treatment of endoscopic submucosal dissection in Japanese: comparison of H_2_ receptor antagonist and proton pump inhibitor administration. *Journal of Clinical Biochemistry and Nutrition*.

[86] Peghini PL, Katz PO, Castell DO (1998). Ranitidine controls nocturnal gastric acid breakthrough on omeprazole: a controlled study in normal subjects. *Gastroenterology*.

[87] Katsube T, Adachi K, Kawamura A (2000). Helicobacter pylori infection influences nocturnal gastric acid breakthrough. *Alimentary Pharmacology and Therapeutics*.

[88] Xue S, Katz PO, Banerjee P, Tutuian R, Castell DO (2001). Bedtime H2 blockers improve nocturnal gastric acid control in GERD patients on proton pump inhibitors. *Alimentary Pharmacology and Therapeutics*.

[89] Wolfe MM, Soll AH (1988). The physiology of gastric acid secretion. *The New England Journal of Medicine*.

